# Old-Fashioned Technology in the Era of “Bling”: Is There a Future for Text Messaging in Health Care?

**DOI:** 10.2196/16630

**Published:** 2019-12-20

**Authors:** Jane C Willcox, Rosie Dobson, Robyn Whittaker

**Affiliations:** 1 School of Allied Health, Human Services and Sport La Trobe University Bundoora Australia; 2 National Institute for Health Innovation University of Auckland Auckland New Zealand; 3 Waitemata District Health Board Auckland New Zealand

**Keywords:** text messaging, mHealth, behavior change, digital health

## Abstract

In the quest to discover the next high-technology solution to solve many health problems, proven established technologies are often overlooked in favor of more “technologically advanced” systems that have not been fully explored for their applicability to support behavior change theory, or used by consumers. Text messages or SMS is one example of an established technology still used by consumers, but often overlooked as part of the mobile health (mHealth) toolbox. The purpose of this paper is to describe the benefits of text messages as a health promotion modality and to advocate for broader scale implementation of efficacious text message programs. Text messaging reaches consumers in a ubiquitous real-time exchange, contrasting the multistep active engagement required for apps and wearables. It continues to be the most widely adopted and least expensive mobile phone function. As an intervention modality, text messaging has taught researchers substantial lessons about tailored interactive health communication; reach and engagement, particularly in low-resource settings; and embedding of behavior change models into digital health. It supports behavior change techniques such as reinforcement, prompts and cues, goal setting, feedback on performance, support, and progress review. Consumers have provided feedback to indicate that text messages can provide them with useful information, increase perceived support, enhance motivation for healthy behavior change, and provide prompts to engage in health behaviors. Significant evidence supports the effectiveness of text messages alone as part of an mHealth toolbox or in combination with health services, to support healthy behavior change. Systematic reviews have consistently reported positive effects of text message interventions for health behavior change and disease management including smoking cessation, medication adherence, and self-management of long-term conditions and health, including diabetes and weight loss. However, few text message interventions are implemented on a large scale. There is still much to be learned from investing in text messaging delivered research. When a modality is known to be effective, we should be learning from large-scale implementation. Many other technologies currently suffer from poor long-term engagement, the digital divide within society, and low health and technology literacy of users. Investing in and incorporating the learnings and lessons from large-scale text message interventions will strengthen our way forward in the quest for the ultimate digitally delivered behavior change model.

## Background

Text messaging is old fashioned, let’s use Facebook instead.

Health researcher, 2018

The evolution of digital technologies, and ubiquity of personal mobile phones, has seen a proliferation in potential options for digital health delivery. The quest among health care organizations is to discover the next high-technology solution to many of the health problems that have been vexing health professionals and researchers for decades.

The increasing availability of consumer-accessible technologies, along with the expectations of an increased consumer role in managing their own health, is contributing to the increased digitalization of health care [1]. Moreover, financial constraints on expensive health services, including the decreased time health providers have in direct patient-provider interaction [1], is changing the health education and information delivery paradigm. The significant increase in digital health investment by the commercial industry [2] is moving the trusted voice of the health providers to the open market. For example, only 10% of health smartphone apps available in major app stores are produced by universities, nongovernment organizations, and educational organizations [2]. To participate in this market, researchers and health providers need to find ways to digitally connect with consumers in a trusted, meaningful, and cost-effective manner.

Many digital health companies and researchers are attempting to find the next neatly packaged behavior change app or device. As academics, we would like to think that we are immune to fashion and trends, but are we? Craig Fleming [3] notes in his provocative essay, “The Tyranny of Trendy Ideas: Academics pretend to be above cheap and trivial fads,” that the drive of “innovation” tends to move academic groups because fashions are “difficult to resist,” but this prevents us from fully investigating phenomena before moving onto the next idea and “distract(s) us from slower changes.” Too often, the focus of research is on the digital delivery modality driving the intervention, often neglecting the complex nature of the behavior change on which it is designed to focus.

Health-related behaviors and behavioral risk factors for disease prevention and management are complex, influenced by multiple individual, socioeconomic, societal, cultural, and environment factors, making it difficult to change [4]. Digital health technologies allow us to accommodate many of these factors with significant potential for digital health solutions to better support prevention and management of disease. Health behavior science provides insight into factors that influence specific actions that can be used to guide digital health design [5]. There are many simple digital technologies that connect directly to consumers and offer benefits for behavior change but have been discarded by funders, health services, and researchers because they are not considered “innovative” or “shiny” enough. Research on proven, more established technologies is often overlooked in favor of what is more “technologically advanced,” even if the use of the new technology is not fully explored, not underpinned by behavior change theory, and not routinely used by or considered of value to the health consumer. Text messages (or SMS) are one example of an established technology, frequently used by consumers, that is often overlooked by many researchers and funders as part of their mobile health (mHealth) toolbox. Often, these authors have been told by funders that it is proven that text messaging works, but it is just “not shiny enough” for their boards, donors, clinicians, or consumers.  

Text messaging is the real-time exchange of alphanumeric messages of up to 160 characters via mobile phones or computer. Text messaging is ubiquitous and continues to be the most widely adopted and least expensive technological function on mobile phones [6]. **The use of d**irect text messaging has remained constant despite the exponential rise of message apps [7]. Paraphrasing Mark Twain [8], the prediction of the death of text messages has been greatly exaggerated. Although digital health innovators may be discarding text messaging for its “low” technology, text messaging interventions have underpinned a significant piece of the mHealth research landscape. It behooves us to examine what text messages and their interventions offer us as researchers and what lessons may be taken forward and incorporated into new and disruptive technologies.

The purpose of this paper is two-fold. First, we describe the present-day benefits of text messages as a health promotion modality, such as high consumer familiarity and usage, functionality to prompt behavior change, and ability to reach hard-to-reach populations. Second, we advocate for broader-scale implementation of efficacious text message programs and continued research to refine and enhance the impact of text messages for health promotion. In this paper, we use quotes and colloquial language communicated to us in research studies and by funders and fellow researchers, to contextualize current-day dialogue concerning the use of text messaging within the research and implementation science research environment.

## Text Messages Connect Directly With Consumers Through a Familiar Modality That They Frequently Use

They will always check their text messages in an appointment, they prioritise them over me.

Health professional, 2016

A digital health intervention program needs to be easy to access...but there’s so many things you have to login so much yeah passwords…whereas if…it’s just link to the website or game in a text, like it just took you straight there rather than, oh I need to remember to check that website or app once every week.

Focus group research participant, 2016

Text messages are elegant in their simplicity and connectivity. They offer many advantages over other digital modalities and are currently the most expeditious way to provide just-in-time information. Text messages are a “push” technology, where intervention messages are delivered to individuals without any effort from the individual [9] and exhibit a 98% open rate and a response rate double that of email, phone, or social media [10].

In contrast, technologies such as apps or games require active participation, that is, the user is required to download the technology, open it, and do something (eg, add data). Moreover, an app or game may be deleted and the automated functions, such as push notifications, can be turned off, or the linked wearable sensors may be not be worn [11,12]. Much like internet health programs, apps only work for those who actively engage with them over a period of time and exhibit the necessary level of digital literacy. More work is required to develop “sticky” apps, which induce return traffic and maintain user engagement, such as Google Maps, that are used most days on smartphones [13,14].

The simple digital user interface for text messaging offers a communication platform that a majority of consumers are comfortable and familiar with. Although it is acknowledged that more work is required to better understand message requirements for those with physical disabilities or low literacy [15], the interface is standardized and reduces the need for learning interaction with a new interface. In contrast, complex digital tools (including apps, wearables, and games) encompass the technological device and the technology interface and performance (interface design, navigation, notifications, data collection methods and tools, goal management, depth of knowledge, system rules, and actionable recommendations) [16], exposing a complex phenomenological structure for user engagement and perception [17]. The lack of a user-centric design is a common criticism of more complex technologies [17]. How consumers engage and perceive digital health interventions still requires much more investigation but, in general, the simpler the interface is, the easier it is to engage and retain consumers [17-19].

## Text Messages Directly Support Behavior Change

Text messaging is just a few words sent to someone. Who is going to send them? Too much money and bother. We want to spend money on something much more impressive such as an app or a robot.

Public health manager/researcher, 2018

As an intervention modality, text messaging has taught researchers substantial lessons about tailored, interactive, and scalable health communication; reach and engagement; and embedding of behavior change models into digital health. Text messaging appears to support behavior change through the ease of application of proven behavior change techniques such as reinforcement, prompts and cues, goal setting, feedback on performance, support, and progress review [20]. The interactivity of text messages allows individuals to log their health information in response to text messages and, in turn, receive tailored feedback. They also have the potential to provide two types of “sticky” content or content that induces return engagement and holds user attention [14]. They can deliver attracting (such as health information updates) and “entrapping” (such as behavioral reminders and bidirectional engagement with a health team) content, which research suggests attracts users and keeps them engaged with a digital platform [13,14].

Text messaging works alone or as a component of an mHealth toolbox. Consumers are seeking simple and intuitive digital solutions to support their health care management [21,22]. In research contexts, individual digital health interventions are often used alone and compared with others, instead of considering how best to meet holistic needs with more effective and efficient health care [23]. It is likely that the digital solutions to complex health behavior change will be multifaceted with the need to draw on the communication, behavioral, and human-computer interaction theory [19]. Real-world needs are likely to be met by a range of interoperable digital platforms or tools integrated with brick-and-mortar health services. Text messaging can be part of this solution, offering many facets to a mobile health (mHealth) toolbox where otherwise high attrition rates, digital divide of society, and the health and technological literacy of the users are key issues.

Although text messaging has proven to be effective when used singularly, evidence suggests that text interventions were efficacious when combined with supplementary intervention components [24,25]. Text messages offer opportunities to optimize interventions and link between components. For example, in one of our studies involving multiple digital components, the most frequently accessed Web pages and Youtube videos were those linked to a directly delivered text message [26]. They also offer a “nudging” capability as per Pew Research Center [27], demonstrating an improved response time for survey completion with text message notifications in comparison to email only [27]. Opportunity exists to exploit text messaging features for better uptake and engagement of other digital health tools when systematically assessing the digital tool delivery required to meet consumer health needs [28]. Although many moderators at a behavioral level require confirmation, we need to still learn about the frequency of contact, wording, content, tone of delivery, and personal tailoring of the short messages [29]. There is potential value in systematically assessing these moderators to allow the learnings to be incorporated into newer technology tools and interventions.

## Text Messages Have the Potential to Reduce Health Inequities at a Low Cost

It’s capturing the people that miss out…the marginalised people that always slip through the system, you can catch them.

Midwife, 2016

If our role as health researchers is to positively impact those who carry the disproportionate burden of poor health outcomes [30], it is crucial for us to consider which digital platforms may best serve those in greatest need. Mobile phones have been widely adopted among virtually all demographic groups, including previously difficult-to-reach populations [29,31-33]. However there remains a digital access disconnect and a usage gap between groups of users with access to the internet (data plans) and the use of smartphone apps that require a greater burden on the consumer. Even in text messaging, there can be difficulty with literacy [15], and more work is required to engage particularly those with physical and intellectual disabilities. The intricacy of more complex digital interactions and expectations may work against accessibility in many population groups.

The appeal and efficacy of text messaging is demonstrated across age, sociodemographic status, and cultures [33,34]. Of note, text messaging interventions can appeal to, connect with, and achieve positive health outcomes for the most difficult-to-reach communities including those that do not connect with traditional health services [35,36]. Due to the ease of tailoring text messaging interventions, programs can be delivered in multiple languages, locations, and cultural versions to ensure relevance and appropriateness for a wide range of populations [32,37].

Although text messaging may be considered relatively expensive to deliver due to “per text message” costs and short code fees, text messaging programs are cheaper to develop: The cost of the average commercial health app (the consumer expected standard of app) is around US $425,000, and higher costs are generally associated with better design, resulting in higher engagement [2]. Furthermore, text messaging can be received at no cost to the end user regardless of phone type, capacity, or data access. Where needed, reply messaging can be charged back to the program to ensure no cost to the recipient and therefore reduce engagement barriers. There is little recognition of the ongoing upkeep and maintenance needed for other digital tools; for example, smartphone apps require updates for every operating system update. These maintenance and development costs have had a negative impact on the business cases for these tools.

The uptake of text messaging in developing countries suggests opportunities in resource-poor settings where expensive technology and internet access is lacking [38]. There is already evidence of the potential health uplift in developing countries where text messages have been utilized in smoking cessation, antenatal, diabetes, and retroviral interventions as well as communication with health workers [39-41].

## Text Message Interventions Demonstrate Effectiveness in Health Promotion and Disease Management

Text messaging works but it’s not shiny enough. We need an app.

Health researcher, 2017

Publication of text message─delivered behavioral intervention studies started to emerge in the early 2000s, gaining traction over the last decade [42], which is a long time in terms of agile technology but not in evidence-based, academic literature. There is significant evidence to support the effectiveness of text messaging alone as part of an mHealth toolbox or in combination with face-to-face health services, to support healthy behavior change. Systematic reviews and meta-analyses have consistently reported positive effects of text message interventions for health behavior change or promotion [25,42-46] and disease management [43,45,47,48]. High-quality evidence may be found across health issues for smoking cessation [24], medication adherence [49] including antiretroviral therapy [47,50], and self-management of long-term conditions and health including diabetes and weight loss [42,46,51,52]. For example, the latest Cochrane review of 26 smoking cessation studies (n=33,849) provides continued evidence for automated app-based interventions resulting in improved cessation rates (increasing quit rates by 50%-60%), while highlighting the persistence of a lack of evidence to conclude the effectiveness of smoking cessation appb─ased interventions [24].

In contrast, systematic reviews of smartphone apps have thus far reported mainly pilots and studies with small sample sizes with limited evidence of effectiveness [24,53,54], although their application in mental health [55], schizophrenia [56] and weight loss [57] is promising. The majority of the 300,000 health apps in app stores have never been tested, and many lack the behavior theory or clinical guidelines to underpin their education frameworks and content [58]. Several research trials have shown apps to be ineffective in achieving significant primary outcomes [59-61]. The lack of a body of evidence for effectiveness currently limits the “prescribablity” of health apps [22,62].

These effective text message interventions provide insights regarding other important aspects of study design including feasibility and target group acceptance. The high levels of retention, acceptability, feasibility, and likelihood of recommending the interventions to peers reported in many studies emphasize the value of using text messaging in health behavior change interventions [26,36,63]. Participants frequently report that they think that text messaging is a good way to deliver these types of prompts, information, and support [32,36,64,65].

Long-term intervention effectiveness and cost-effectiveness studies for all digital interventions remain scarce, and text messaging interventions may offer some guidance and learnings [24,25]. Our recent 2-year follow-up of a text messaging diabetes self-management support program found sustained improved results following the initial trial [66], but that is not always the case, and long-term follow-up needs to be encouraged and further investigated. With the low cost of delivery, smoking cessation support by text messaging has been shown to possibly be one of the most cost-effective health services that could be provided [67,68]. Free and colleagues [67] recruited, via community advertising, smokers willing to quit (n=5800) and randomized them to either the txt2stop intervention, comprising motivational messages and behavioral change support, or the control group that received text messages unrelated to quitting. Biochemically verified smoking cessation rates at 6 months were doubled in the txt2stop group (10.7% in the txt2stop group vs 4.9% in the control group; risk ratio: 2.20; 95% CI 1.80-2,68; *P*<.001). The cost-effectiveness analysis found that when future health service costs were included, text message─based smoking cessation support would save costs to the national health service. Few other cost-effectiveness analyses have been published. Further research is required to determine how to best optimize outcome and cost effectiveness in larger and longer-term trials.

## Implementation of Large-Scale Text Messaging Programs is Required

That’s great that the program was found to be effective but why hasn’t it been rolled out yet?

Clinician, 2019

Implementation of research findings is an ongoing problem for health-related research [69]. The translation and implementation of technology-driven interventions have the added pressure of convincing funders and health bodies that the technology will remain relevant and viable [70]. Although significant evidence for text messaging interventions exists, few large-scale roll outs have been realized or evaluated. There are a small number of examples of smoking cessation interventions proven in research that have been implemented at a large scale internationally [71-73] and offer learnings. The Indian government has implemented a national mobile cessation (smoking cessation) and mobile diabetes (diabetes control) program promoted through their national email network and using the free “missed call” system for people to register [71]. Study process lessons were learned from the evaluation of that program: A large number of nonsmokers initially registered, due to promotion and the ease of registration, and a large number subsequently dropped out due to the high burden of evaluation questions, which has since been removed.

More work is required both to optimize the effectiveness of text message interventions and to build conceptual frameworks for larger-scale implementation. Through its *Be He@lthy, Be Mobile* program [74], the World Health Organization is supporting the establishment of large-scale text message programs in developing countries from a range of regions and income levels, tackling health issues relevant to the country, from cervical cancer awareness to smoking cessation [71]. This group provides toolkits, expert assistance, international connections, and programs as well as links to more general advice on aspects such as frameworks for prioritization, monitoring, and evaluation [75,76].

Although translational research has become a focus for many, there remain roadblocks in implementing research on a large scale, especially while, as Wolf [77] notes, “spectacular new devices are more fascinating to the public and more lucrative for industry.” The study of implementation science in the digital health field would be enhanced by funding and implementing large-scale community-centered digital models within the world of practice. This will allow the identification of crucial activities and delivery methods for conducting an intervention tailored to the unique needs and contexts of different at‐risk populations and health agencies and further the field of implementation science in the digital space [78]. These could include the key components within the study environment that affect the intervention’s primary outcome, changes in outcome variables over time, and potential confounders external to the study environment [79].

## Conclusions

When it comes to digital interventions, evidence supporting the use of text messaging for health behavior change is substantial. In addition to a wide population reach, text messaging is relatively low cost, can be individually tailored, can be delivered anywhere, is appropriate for low digital literacy, and allows instant delivery and feedback. Text messages are omnipresent in the lives of consumers and offer a simple and connected gateway to encourage positive health behaviors. Their effectiveness in supporting health behavior change across population groups and health areas has not yet been paralleled by any other technologies. For digital platforms to assist consumers with positive health behavior change, the appropriate mix of digital delivery needs to be understood and tested. Text messages offer a subset of features that should be considered in any approach. If health organizations have the courage, there is opportunity and value in implementing text messaging interventions, alone or as part of an mHealth toolbox, and learning from their engagement with consumers. These learnings can then be transferred to new effective technologies as they emerge. Health researchers and health organizations can own this simple technology and control it until newer “shinier” technologies are developed, which can replace the widespread connectivity and persistence of a text message.

## Abbreviations

mHealth: mobile health

## References

[1] Timimi FK (2012). Medicine, morality and health care social media. BMC Med.

[2] R2G: Research2Guidance.

[3] Fleming C The Chronicle Review.

[4] Wilkinson R, Marmot M (2003). World Health Organization.

[5] Michie S, van Stralen MM, West R (2011). The behaviour change wheel: a new method for characterising and designing behaviour change interventions. Implement Sci.

[6] Burke K Text request.

[7] Burke K (2019). Text request.

[8] (2018). Subtropic Publications.

[9] Klasnja P, Pratt W (2012). Healthcare in the pocket: mapping the space of mobile-phone health interventions. J Biomed Inform.

[10] Dobrilova T (2019). Techjury.

[11] Clawson J, Pater J, Miller A, Mynatt E, Mamykina L (2015). No longer wearing: investigating the abandonment of personal health-tracking technologies on craigslist.

[12] Patel MS, Asch DA, Volpp KG (2015). Wearable Devices as Facilitators, Not Drivers, of Health Behavior Change. JAMA.

[13] Viswanathan V, Hollebeek LD, Malthouse EC, Maslowska E, Jung Kim S, Xie W (2017). The Dynamics of Consumer Engagement with Mobile Technologies. Service Science.

[14] Kominers S (2009). Sticky content and the structure of the commercial web.

[15] Nouri SS, Avila-Garcia P, Cemballi AG, Sarkar U, Aguilera A, Lyles CR (2019). Assessing Mobile Phone Digital Literacy and Engagement in User-Centered Design in a Diverse, Safety-Net Population: Mixed Methods Study. JMIR Mhealth Uhealth.

[16] Vaghefi I, Tulu B (2019). The Continued Use of Mobile Health Apps: Insights From a Longitudinal Study. JMIR Mhealth Uhealth.

[17] Torous J, Nicholas J, Larsen ME, Firth J, Christensen H (2018). Clinical review of user engagement with mental health smartphone apps: evidence, theory and improvements. Evid Based Mental Health.

[18] O'Connor Siobhan, Hanlon P, O'Donnell Catherine A, Garcia S, Glanville J, Mair FS (2016). Understanding factors affecting patient and public engagement and recruitment to digital health interventions: a systematic review of qualitative studies. BMC Med Inform Decis Mak.

[19] Suffoletto B (2016). Text Message Behavioral Interventions: From Here to Where?. Curr Opin Psychol.

[20] Michie S, Ashford S, Sniehotta FF, Dombrowski SU, Bishop A, French DP (2011). A refined taxonomy of behaviour change techniques to help people change their physical activity and healthy eating behaviours: the CALO-RE taxonomy. Psychol Health.

[21] Willcox JC, van der Pligt P, Ball K, Wilkinson SA, Lappas M, McCarthy EA, Campbell KJ (2015). Views of Women and Health Professionals on mHealth Lifestyle Interventions in Pregnancy: A Qualitative Investigation. JMIR Mhealth Uhealth.

[22] Mendiola MF, Kalnicki M, Lindenauer S (2015). Valuable Features in Mobile Health Apps for Patients and Consumers: Content Analysis of Apps and User Ratings. JMIR mHealth uHealth.

[23] Becker S, Miron-Shatz T, Schumacher N, Krocza J, Diamantidis C, Albrecht U (2014). mHealth 2.0: Experiences, Possibilities, and Perspectives. JMIR mHealth uHealth.

[24] Whittaker R, McRobbie H, Bullen C, Rodgers A, Gu Y, Dobson R (2019). Mobile phone text messaging and app-based interventions for smoking cessation. Cochrane Database of Systematic Reviews.

[25] Armanasco A, Miller Y, Fjeldsoe B, Marshall A (2017). Preventive Health Behavior Change Text Message Interventions: A Meta-analysis. Am J Prev Med.

[26] Willcox J, Wilkinson S, Lappas M, Ball K, Crawford D, McCarthy E, Fjeldsoe B, Whittaker R, Maddison R, Campbell K (2017). A mobile health intervention promoting healthy gestational weight gain for women entering pregnancy at a high body mass index: the txt4two pilot randomised controlled trial. BJOG.

[27] (2016). Pew Research Center Methods.

[28] Graham AL, Jacobs MA, Cohn AM, Cha S, Abroms LC, Papandonatos GD, Whittaker R (2016). Optimising text messaging to improve adherence to web-based smoking cessation treatment: a randomised control trial protocol. BMJ Open.

[29] Lester RT (2013). Ask, Don't Tell — Mobile Phones to Improve HIV Care. N Engl J Med.

[30] Ball K (2015). Traversing myths and mountains: addressing socioeconomic inequities in the promotion of nutrition and physical activity behaviours. Int J Behav Nutr Phys Act.

[31] Aranda-Jan CB, Mohutsiwa-Dibe N, Loukanova S (2014). Systematic review on what works, what does not work and why of implementation of mobile health (mHealth) projects in Africa. BMC Public Health.

[32] Dobson R, Whittaker R, Bartley H, Connor A, Chen R, Ross M, McCool J (2017). Development of a Culturally Tailored Text Message Maternal Health Program: TextMATCH. JMIR Mhealth Uhealth.

[33] Whittaker R, Umali E, Tanielu H, McCool J (2019). TXTTaofiTapaa: pilot trial of a Samoan mobile phone smoking cessation programme. Journal Global Health Reports.

[34] Cole-Lewis H, Kershaw T (2010). Text messaging as a tool for behavior change in disease prevention and management. Epidemiol Rev.

[35] Grutzmacher SK, Braunscheidel Duru E, Speirs KE, Worthington L, Munger AL, Lachenmayr LA (2017). Using text messages to engage low-income parents in school-based nutrition education. Journal of Hunger & Environmental Nutrition.

[36] Dobson R, Whittaker R, Jiang Y, Maddison R, Shepherd M, McNamara C, Cutfield R, Khanolkar M, Murphy R (2018). Effectiveness of text message based, diabetes self management support programme (SMS4BG): two arm, parallel randomised controlled trial. BMJ.

[37] Bramley D, Riddell T, Whittaker R, Corbett Tim, Lin Ruey-Bin, Wills Mary, Jones Mark, Rodgers Anthony (2005). Smoking cessation using mobile phone text messaging is as effective in Maori as non-Maori. N Z Med J.

[38] Carrillo-Larco RM, Jiwani SS, Diez-Canseco F, Kanter R, Beratarrechea A, Irazola V, Ramirez-Zea M, Rubinstein A, Martinez H, Miranda JJ, GISMAL Group (2018). Implementation Tells Us More Beyond Pooled Estimates: Secondary Analysis of a Multicountry mHealth Trial to Reduce Blood Pressure. JMIR Mhealth Uhealth.

[39] Agarwal S, Perry HB, Long L, Labrique AB (2015). Evidence on feasibility and effective use of mHealth strategies by frontline health workers in developing countries: systematic review. Trop Med Int Health.

[40] Kurumop SF, Bullen C, Whittaker R, Betuela I, Hetzel MW, Pulford J (2013). Improving health worker adherence to malaria treatment guidelines in Papua New Guinea: feasibility and acceptability of a text message reminder service. PLoS One.

[41] Déglise Carole, Suggs LS, Odermatt P (2012). SMS for disease control in developing countries: a systematic review of mobile health applications. J Telemed Telecare.

[42] Head KJ, Noar SM, Iannarino NT, Grant Harrington N (2013). Efficacy of text messaging-based interventions for health promotion: a meta-analysis. Soc Sci Med.

[43] Free C, Phillips G, Galli L, Watson L, Felix L, Edwards P, Patel V, Haines A (2013). The effectiveness of mobile-health technology-based health behaviour change or disease management interventions for health care consumers: a systematic review. PLoS Med.

[44] Militello L, Kelly S, Melnyk B (2012). Systematic review of text-messaging interventions to promote healthy behaviors in pediatric and adolescent populations: implications for clinical practice and research. Worldviews Evid Based Nurs.

[45] Hall AK, Cole-Lewis H, Bernhardt JM (2015). Mobile text messaging for health: a systematic review of reviews. Annu Rev Public Health.

[46] Orr JA, King RJ (2015). Mobile phone SMS messages can enhance healthy behaviour: a meta-analysis of randomised controlled trials. Health Psychol Rev.

[47] Mbuagbaw L, Mursleen S, Lytvyn L, Smieja M, Dolovich L, Thabane L (2015). Mobile phone text messaging interventions for HIV and other chronic diseases: an overview of systematic reviews and framework for evidence transfer. BMC Health Serv Res.

[48] Jones KR, Lekhak N, Kaewluang N (2014). Using mobile phones and short message service to deliver self-management interventions for chronic conditions: a meta-review. Worldviews Evid Based Nurs.

[49] Thakkar J, Kurup R, Laba T, Santo K, Thiagalingam A, Rodgers A, Woodward M, Redfern J, Chow CK (2016). Mobile Telephone Text Messaging for Medication Adherence in Chronic Disease: A Meta-analysis. JAMA Intern Med.

[50] Finitsis DJ, Pellowski JA, Johnson BT (2014). Text message intervention designs to promote adherence to antiretroviral therapy (ART): a meta-analysis of randomized controlled trials. PLoS One.

[51] Saffari M, Ghanizadeh G, Koenig HG (2014). Health education via mobile text messaging for glycemic control in adults with type 2 diabetes: a systematic review and meta-analysis. Prim Care Diabetes.

[52] Siopis G, Chey T, Allman-Farinelli M (2015). A systematic review and meta-analysis of interventions for weight management using text messaging. J Hum Nutr Diet.

[53] Payne HE, Lister C, West JH, Bernhardt JM (2015). Behavioral functionality of mobile apps in health interventions: a systematic review of the literature. JMIR Mhealth Uhealth.

[54] Flores Mateo G, Granado-Font E, Ferré-Grau Carme, Montaña-Carreras Xavier (2015). Mobile Phone Apps to Promote Weight Loss and Increase Physical Activity: A Systematic Review and Meta-Analysis. J Med Internet Res.

[55] Firth J, Torous J, Nicholas J, Carney R, Pratap A, Rosenbaum S, Sarris J (2017). The efficacy of smartphone-based mental health interventions for depressive symptoms: a meta-analysis of randomized controlled trials. World Psychiatry.

[56] Firth J, Torous J (2015). Smartphone Apps for Schizophrenia: A Systematic Review. JMIR Mhealth Uhealth.

[57] Flores Mateo G, Granado-Font E, Ferré-Grau Carme, Montaña-Carreras Xavier (2015). Mobile Phone Apps to Promote Weight Loss and Increase Physical Activity: A Systematic Review and Meta-Analysis. J Med Internet Res.

[58] Chen J, Cade JE, Allman-Farinelli M (2015). The Most Popular Smartphone Apps for Weight Loss: A Quality Assessment. JMIR Mhealth Uhealth.

[59] Ni Mhurchu C, Te Morenga L, Tupai-Firestone R, Grey J, Jiang Y, Jull A, Whittaker R, Dobson R, Dalhousie S, Funaki T, Hughes E, Henry A, Lyndon-Tonga L, Pekepo C, Penetito-Hemara D, Tunks M, Verbiest M, Humphrey G, Schumacher J, Goodwin D (2019). A co-designed mHealth programme to support healthy lifestyles in Māori and Pasifika peoples in New Zealand (OL@-OR@): a cluster-randomised controlled trial. The Lancet Digital Health.

[60] Baskerville NB, Struik LL, Guindon GE, Norman CD, Whittaker R, Burns C, Hammond D, Dash D, Brown KS (2018). Effect of a Mobile Phone Intervention on Quitting Smoking in a Young Adult Population of Smokers: Randomized Controlled Trial. JMIR Mhealth Uhealth.

[61] Laws RA, Denney-Wilson EA, Taki S, Russell CG, Zheng M, Litterbach E, Ong K, Lymer SJ, Elliott R, Campbell KJ (2018). Key Lessons and Impact of the Growing Healthy mHealth Program on Milk Feeding, Timing of Introduction of Solids, and Infant Growth: Quasi-Experimental Study. JMIR Mhealth Uhealth.

[62] Byambasuren O, Sanders S, Beller E, Glasziou P (2018). Prescribable mHealth apps identified from an overview of systematic reviews. NPJ Digit Med.

[63] Speirs KE, Grutzmacher SK, Munger AL, Messina LA (2016). Recruitment and retention in an SMS-based health education program: Lessons learned from Text2BHealthy. Health Informatics J.

[64] Heminger CL, Boal AL, Zumer M, Abroms LC (2016). Text2Quit: an analysis of participant engagement in the mobile smoking cessation program. Am J Drug Alcohol Abuse.

[65] Douglas N, Free C (2013). ‘Someone batting in my corner’: experiences of smoking-cessation support via text message. Br J Gen Pract.

[66] Dobson R, Whittaker R, Jiang Y, McNamara C, Shepherd M, Maddison R, Cutfield R, Khanolkar M, Murphy R (2019). Long-term follow-up of a randomized controlled trial of a text-message diabetes self-management support programme, SMS4BG. Diabet Med.

[67] Free C, Knight R, Robertson S, Whittaker R, Edwards P, Zhou W, Rodgers A, Cairns J, Kenward MG, Roberts I (2011). Smoking cessation support delivered via mobile phone text messaging (txt2stop): a single-blind, randomised trial. The Lancet.

[68] Guerriero C, Cairns J, Roberts I, Rodgers A, Whittaker R, Free C (2013). The cost-effectiveness of smoking cessation support delivered by mobile phone text messaging: Txt2stop. Eur J Health Econ.

[69] Wensing M, Grol R (2019). Knowledge translation in health: how implementation science could contribute more. BMC Med.

[70] Gurupur VP, Wan TTH (2017). Challenges in implementing mHealth interventions: a technical perspective. Mhealth.

[71] Gopinathan P, Kaur J, Joshi S, Prasad VM, Pujari S, Panda P, Murthy P (2018). Self-reported quit rates and quit attempts among subscribers of a mobile text messaging-based tobacco cessation programme in India. BMJ Innov.

[72] Abroms LC, Johnson PR, Leavitt LE, Cleary SD, Bushar J, Brandon TH, Chiang SC (2017). A Randomized Trial of Text Messaging for Smoking Cessation in Pregnant Women. Am J Prev Med.

[73] Rodgers A, Corbett T, Bramley D, Riddell T, Wills M, Lin R-b, Jones M (2005). Do u smoke after txt? Results of a randomised trial of smoking cessation using mobile phone text messaging. Tob Control.

[74] (2019). World Health Organization.

[75] World Health Organization.

[76] (2019). WHO Guideline: recommendations on digital interventions for health system strengthening.

[77] Woolf SH (2008). The meaning of translational research and why it matters. JAMA.

[78] Wandersman A, Duffy J, Flaspohler P, Noonan Rita, Lubell Keri, Stillman Lindsey, Blachman Morris, Dunville Richard, Saul Janet (2008). Bridging the gap between prevention research and practice: the interactive systems framework for dissemination and implementation. Am J Community Psychol.

[79] McAlearney AS, Walker DM, Livaudais-Toman J, Parides M, Bickell NA (2016). Challenges of implementation and implementation research: Learning from an intervention study designed to improve tumor registry reporting. SAGE Open Med.

